# Genetic parameters and selection in full-sib families of tall fescue using best linear unbiased prediction (BLUP) analysis

**DOI:** 10.1186/s12870-022-03675-w

**Published:** 2022-06-14

**Authors:** Fatemeh Pirnajmedin, Mohammad Mahdi Majidi, Mohammad Hadi Taleb, Davoud Rostami

**Affiliations:** 1 Department of Agronomy and Plant Breeding, College of Agriculture, Isfahan University of Technology, Isfahan, 84156-83111 Iran; 2 Department of Animal Science, College of Agriculture, Isfahan University of Technology, Isfahan, 84156-83111 Iran

**Keywords:** Breeding value, Full-sib, Inheritance, REML/BLUP, Tall fescue

## Abstract

**Background:**

Better understanding of genetic structure of economic traits is crucial for identification and selection of superior genotypes in specific breeding programs. Best linear unbiased prediction (BLUP) is the most efficient method in this regard, which is poorly used in forage plant breeding. The present study aimed to assess genetic variation, estimate genetic parameters, and predict breeding values of five essential traits in full sib families (recognized by EST-SSR markers) of tall fescue using REML/BLUP procedure.

**Method:**

Forty-two full-sib families of tall fescue (included of 120 individual genotypes), recognized by EST-SSR markers along with twenty-one their corresponding parental genotypes were assessed for biomass production and agro-morphological traits at three harvests (spring, summer, and autumn) in the field during 4 years (2017–2020).

**Results:**

Considerable genotypic variability was observed for all traits. Low narrow-sense heritability (h^2^_n_) for dry forage yield (DFY) at three harvest indicates that non-additive gene actions may play an important role in the inheritance of this trait. Higher h^2^_n_ of yield related traits and flowering time and also significant genetic correlation of these traits with forage yield, suggests that selection based on these traits may lead to indirect genetic improvement of DFY.

**Conclusion:**

Our results showed the adequacy of REML/BLUP procedure for identification and selection of preferable parental genotypes and progenies with higher breeding values for future breeding programs such as variety development in tall fescue. Parental genotypes 21 M, 1 M, and 20 L were identified as superior and stable genotypes and could also produce the best hybrid combinations when they were mostly used as maternal parent.

**Supplementary Information:**

The online version contains supplementary material available at 10.1186/s12870-022-03675-w.

## Background

Climate change and desertification offer several biotic and abiotic stresses, limiting growth and production of plants in many areas of the world [1, 2]. As a result, the primary forage breeder goal is to select perennial plants species or genotypes that are able to have suitable and stable productivity over several years [3, 4].

Tall fescue (*Festuca arundinacea* Schreb. syn. *Lolium arundinaceum*) is known as an important cool-season perennial grass, widely used for forage and turf application due to its persistence, notable local adaptation, forage production, and tolerance to biotic and abiotic stresses [5–7]. It is an allohexaploid (2n = 6x = 42), self-incompatible, out-crossing species and cultivars are usually produced through random mating of several selected parents using the polycross method resulting in population-based synthetic [8].

Selecting the right parents for create new genetic variants and choosing preferable recombinants or favorable progenies with desire traits are critical steps in the breeding programs [9, 10]. Therefore, knowledge on the genetic diversity, heritable variations, and genetic correlation among the selection traits is essential for selecting superior genotypes and improving the efficiency of breeding programs [11, 12]. In grass species, most of the economical traits such as forage or biomass yield are genetically complex traits with quantitative inheritance and affected by the genotype and environment interaction [13, 14]. The interaction of genetic by year can limit the yield stability and selection of superior genotypes, so assessment of genotypes in different years is one of the important parts of the breeding programs [13, 15].

The utility of incorporating pedigree information in the evaluation of plant production has been illustrated in some plant species [16, 17]. Integrating genetic relations based on pedigree information increases the precision prediction in breeding experiments, helping to distinguished desirable parent genotypes for future crosses, as well as detect promising families or progenies for commercial expansion [9, 16, 18].

The use of restricted maximum likelihood (REML) followed by the best linear unbiased prediction (BLUP) is the most efficient method, for estimation of genetic parameters and prediction breeding values and identification of favorable individuals in the selected breeding populations [17, 19]. The use of BLUP generate accurate for estimation of genetic parameters and prediction of genotypic values (additive effects) even in unbalanced experimental designs, and it’s also can use to estimate genetic correlations among performance of the same genotypes in different conditions [17, 20, 21]. Abu-Ellail et al. [20] used the BLUP method to evaluate 19 sugarcane families at single stool stage of breeding program, estimate genetic parameters, and predict genetic values by analysis individuals within families. Asfaw et al. [9] confirmed that the BLUP procedure had great efficiency in selection of superior parental clones and progenies with higher genotypic and breeding values for cultivar development in yam. Acharya et al. [15] used the BLUP method in alfalfa to generate information concerning genetic parameters, obtain genotypes rankings, and select families with preferable agronomic traits.

Breeding of cross-pollinated grasses such as tall fescue is very difficult due to their high degree of self-incompatibility and cleistogamous flowers [22]. In this kind of grasses half-sib mating including open-pollination, top cross, and polycross are common and cost effective breeding procedures for creating a basic pool of genetic variation [23]. However, in half-sib families derived from these breeding procedures recognition of pedigree information and kinship relationships will be very difficult, because the maternal parent is preserved, but paternal parent is usually unknown [24]. Due to this event, reconstructing the pedigree of the target progeny for genetic improvement and identification the paternal parent will be possible using molecular markers [25]. In our previous research, we constructed a ploycross breeding population in tall fescue and reconstructed the pedigree of the target half-sib progenies using EST-SSR molecular markers for paternity identification and finding full-sib families [26]. Nevertheless, still little information is available on the application of BLUP procedure for estimating genetic parameters and breeding values in forage plant breeding specially, in full-sib families of tall fescue. Therefore, in the present study we aimed to (1) assess the genetic diversity of full-sib families of tall fescue (recognized by EST-SSR markers from a big progeny nursery) for biomass production and agro-morphological traits during four consecutive years, (2) estimate genetic parameters and predict breeding value of measured traits using REML/BLUP procedure, and subsequently select full-sib families and elite individual clones with higher breeding values and yield stability.

## Results

Analysis of variance indicated the existence of significant genetic variation (*P* < 0.01) between the parental genotypes and full-sib families of tall fescue across three harvests for all the measured traits (Table S1). The effect of harvest and year were also significant for all traits (Table S1). Mean of dry forage yield ranged from 37.80 to 165.74 g/plant in the parental genotypes and from 73.16 to 352.41 g/plant in the full-sib families (Table 1). Parental genotypes 20 L and 21 M and full-sib families 1, 26, and 41 had the highest values of dry forage yield and the lowest values belonged to parental genotypes 22 M and 3E and full-sib families 6, 29, and 36. The stability parameter based on regression method (b-value) ranged from 0.45 to 2.26 in parental genotypes and from 0.46 to 2.06 in full-sib families. Parental genotypes 21 M, 23 M, 15 L, and 1 M and full-sib families 25, 34, 38, 41, 26, 39, 19, and 12 with regression coefficients for forage yield close to unity can be considered to display high yield stability across years.

**Table 1:** Information about name, mean dry forage yield, and stability parameter of 21 parental genotypes and 42 full-sib families of tall fescue used in this study

Parental genotypes						Full-sib families									
Parental genotype name	Origin	Maternal genotype	Paternal genotype	Mean dry forage yield (g/plant)	Yield stability (bi)	Full-sib family code	Parental plants	Number of genotype in each full-sib family	Mean dry forage yield (g/plant)	Yield stability (bi)	Full-sib family code	Parental plants	Number of genotype in each full-sib family	Mean dry forage yield (g/plant)	Yield stability (bi)
1E	Iran, Isfahan,Yazdabad	Yes	Yes	76.8	1.14	1	♀1E × ♂2E	2	352.41	0.58	22	♀17 M × ♂1 M	7	160	1.18
2E	Iran,Yasuj	No	Yes	87.24	0.54	2	♀1E × ♂4E	1	223.91	0.51	23	♀17 M × ♂2 L	1	292.7	0.51
3E	Iran, Isfahan, Mobarake	Yes	No	39.45	1.86^a^	3	♀1E × ♂1 M	6	135.54	1.46	24	♀21 M × ♂14E	2	158.72	1.24
4E	Iran, Isfahan, Mobarake	Yes	Yes	49.76	1.65^a^	4	♀3E × ♂10E	2	159.37	1.20	25	♀21 M × ♂1 M	3	200.69	0.93
10E	USA, New Jersy	Yes	Yes	41.47	2.09^a^	5	♀3E × ♂14E	2	178.31	1.08	26	♀21 M × ♂11 M	2	307.74	0.96
14E	Hungary, unknown	Yes	Yes	88.26	0.81	6	♀3E × ♂1 M	1	73.16	1.91^a^	27	♀21 M × ♂6 L	1	190.62	0.46
16E	Iran, Isfahan, Fozve	No	Yes	77.54	0.55	7	♀3E × ♂11 M	3	149.16	1.31	28	♀22 M × ♂4E	2	144.45	1.26
1 M	Iran, Isfahan,Yazdabad	Yes	Yes	132.35	1.02	8	♀4E × ♂11 M	2	294.74	0.57	29	♀22 M × ♂10E	1	92.58	2.06^a^
3 M	Iran, Yasuj	Yes	No	72.27	0.57	9	♀4E × ♂17 M	3	195.9	0.87	30	♀22 M × ♂1 M	2	201.7	0.73
11 M	Hungary, unknown	Yes	Yes	55.67	1.59	10	♀10E × ♂1 M	5	200.23	0.8	31	♀22 M × ♂21 M	1	107.7	1.92^a^
17 M	Iran, Isfahan, Fozve	Yes	Yes	58.57	1.24	11	♀14E × ♂1E	1	146.41	1.14	32	♀23 M × ♂14E	2	290.45	0.55
21 M	Iran, Isfahan, Fozve	Yes	Yes	141.44	0.97	12	♀14E × ♂2E	11	198.3	1.03	33	♀23 M × ♂17 M	2	168.66	1.12
22 M	Poland, unknown	Yes	No	37.8	2.26^a^	13	♀14E × ♂25 L	2	276.55	0.7	34	♀23 M × ♂3 L	2	237.22	0.94
23 M	Poland, unknown	Yes	No	133.52	1	14	♀14E × ♂11 M	6	181.19	1.06	35	♀23 M × ♂15 L	1	194.66	0.66
2 L	Iran, Isfahan, Daran	No	Yes	45.58	1.43	15	♀1 M × ♂21 M	3	148.85	1.13	36	♀6 L × ♂1E	2	97.51	1.94^a^
3 L	Iran, Isfahan, Yasuj	No	Yes	93.85	0.59	16	♀3 M × ♂2E	3	225.66	0.78	37	♀6 L × ♂1 M	1	194.58	0.81
6 L	Iran, Isfahan, Daran	Yes	Yes	129.89	0.45	17	♀3 M × ♂1 M	4	203.19	0.58	38	♀12 L × ♂1E	6	246.66	0.95
12 L	Iran, Isfahan, Daran	Yes	No	117.65	0.66	18	♀3 M × ♂6 L	1	102.83	1.86^a^	39	♀12 L × ♂1 M	4	184.42	0.97
15 L	Hungary, unknown	No	Yes	64.53	1.01	19	♀11 M × ♂2E	9	195.01	1.02	40	♀12 L × ♂2 L	3	287.84	0.64
20 L	Iran, Isfahan, Yazdabad	Yes	No	165.74	0.59	20	♀11 M × ♂16E	1	159.41	1.19	41	♀20 L × ♂4E	4	300.85	0.95
25 L	Iran, Isfahan, Fozve	No	Yes	121.74	0.76	21	♀11 M × ♂1 M	1	130.7	1.22	42	♀20 L × ♂14E	2	288.81	0.59

^a^Significantly different from 1 for bi at the 0.05 probability level

**Fig. 1:**
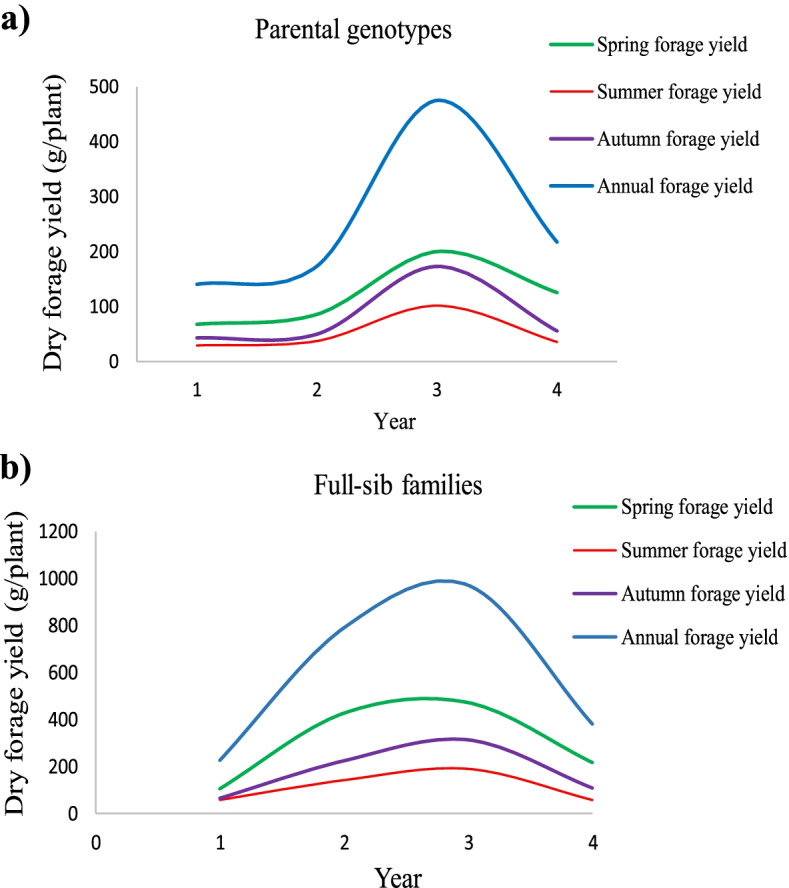
Spring, summer, autumn, and annual dry forage yield in parental genotypes and full-sib families of tall fescue evaluated during 4 years (2017–2020) in the field

In both parental genotypes and full-sib families, the highest forage yield (including SPDFY, SUDFY, AUDFY, and ADFY) was observed in the third year of this experiment compared to the first, second and fourth years (Fig. 1). Mean values of morphological traits for three harvests over 4 years of experiments are presented in Table 2, in which significant differences can be seen between spring, summer, and autumn harvests for dry forage yield (DFY), plant height (H), and crown diameter (CD) in both parental genotypes and full-sib families over years. The highest and lowest values of DFY, H, and CD in both parental genotypes and full-sib families were obtained at the spring and summer harvests during 4 years of experiment, respectively.

**Table 2:** Means of morphological traits in parental genotypes and full-sib families of tall fescue at three harvests (spring, summer and autumn) during 2017–2020

**Parental genotypes**												
	**2017**			**2018**			**2019**			**2020**		
Harvest	DFY (g/plant)	H (cm)	CD (cm)	DFY (g/plant)	H (cm)	CD (cm)	DFY (g/plant)	H (cm)	CD (cm)	DFY (g/plant)	H (cm)	CD (cm)
Spring	67.97^a^	32.23^a^	15.93^a^	86.12^a^	58.48^a^	23.11^a^	200.19^a^	103.40^a^	27.09^a^	125.77^a^	62.45^a^	16.09^a^
Summer	29.46^c^	22.02^b^	9.19^b^	37.62^c^	30.12^b^	15.11^b^	101.85^c^	46.40^c^	18.64^b^	35.92^c^	26.54^c^	5.547^c^
Autumn	43.28^b^	29.79^ab^	9.53^b^	50.07^b^	34.04^b^	17.16^b^	173.21^b^	65.90^b^	23.88^ab^	55.91^b^	45.26^b^	10.38^b^
**Full-sib families**												
	**2017**			**2018**			**2019**			**2020**		
	DFY (g/plant)	H (cm)	CD (cm)	DFY (g/plant)	H (cm)	CD (cm)	DFY (g/plant)	H (cm)	CD (cm)	DFY (g/plant)	H (cm)	CD (cm)
Spring	104.51^a^	48.28^a^	16.01^a^	426.28^a^	76.92^a^	25.53^a^	471.02^a^	117.20^a^	34.72^a^	215.96^a^	85.07^a^	22.34^a^
Summer	57.37^b^	38.53^b^	9.05^b^	140.90^c^	39.20^c^	17.47^b^	188.53^c^	54.92^c^	25.52^b^	57.04^c^	34.78^c^	10.60^c^
Autumn	63.54^b^	42.34^b^	11.50^b^	222.49^b^	53.24^b^	19.28^b^	311.14^b^	80.62^b^	31.95^ab^	106.80^b^	64.70^b^	16.69^b^

*DFY* dry forage yield, *H* plant height, *CD* crown diameter

^a,b,c^In each coulmn, in parental genotypes or full-sib families, means sharing different letter are significantly different at the 5% level by LSD test

Estimations of narrow sense heritability (h^2^_n_) for measured traits and relative selection efficiency (RSE) for improvement of dry forage yield (DFY) in a single harvest and in multiple harvest analysis are given in Tables 3 and 4, respectively. In a single harvest analysis, narrow sense heritability ranged from 0.18 (DFY) to 0.29 (NS) at the spring harvest, and from 0.16 (DFY) to 0.26 (CD) at the summer harvest, and from 0.15 (DFY) to 0.25 (CD) at the autumn harvest (Table 3). Generally, the highest estimates of h^2^_n_ for measured traits were obtained in the spring harvest, whereas the lowest estimates were obtained in the later harvests (summer and autumn). In multiple harvest analysis, low to moderate values of h^2^_n_ were observed for all of the evaluated traits (Table 4), ranging from 0.22 (DFY, FL) to to 0.41 (CD). In both single and multiple harvest analysis, the h^2^_n_of yield related traits consisted of plant height (H), the number of stems per plant (NS), and CD was greater than the h^2^_n_of dry forage yield (Tables 3 and 4). In both single and multiple harvest analysis, the highest values of correlated response and relative selection efficiency (RSE > 1) for genetically improvement of DFY was obtained via selection for H, CD, and NS.

**Table 3:** Estimates of variance components (VC) and narrow sense heritability (h^2^_n_) of measured traits and relative efficiency of indirect selection (RSE) for improvement of DFY in evaluated genotypes of tall fescue in a single harvest analysis

	Spring harvest					Summer harvest			Autumn harvest		
VC	DFY	H	CD	NS	FL	DFY	H	CD	DFY	H	CD
${\sigma}_A^2$	5121.79	99.56	11.43	44.5	11.78	1384.67	35.79	9.7	4102.92	64.52	13.53
${\sigma}_p^2$	28,171.93	366.01	40.67	149.71	47.57	8308.06	141.04	36.6	26,202.41	278.39	52.37
${h}_n^2\pm \mathrm{S}E$	0.18 ± 0.03	0.27 ± 0.03	0.28 ± 0.03	0.29 ± 0.04	0.24 ± 0.04	0.16 ± 0.04	0.25 ± 0.03	0.26 ± 0.04	0.15 ± 0.04	0.23 ± 0.04	0.25 ± 0.05
R_y_	52.87	9.03	3.12	6.20	2.89	25.52	5.19	2.75	42.49	6.71	3.16
CR_y_	–	53.84	54.18	53.14	29	–	24.47	26.02	–	43.88	44.67
RSE	–	1.01	1.02	1	0.54	–	1.03	1.01	–	1.03	1.05

*DFY* dry forage yield (g/plant), *H* plant height (cm/plant), *CD* crown diameter (cm/plant), *NS* number of stems per plant, *FL* Flowering time

σ^2^_A_ additive and σ^2^_p_ phenotypic variance, h^2^_n_ narrow sense heritability, *SE* standard error, *R*_y_ response to selection, *CR*_y_ correlated response to selection

**Table 4:** Estimates of variance components and narrow sense heritability (h^2^_n_) of measured traits and relative efficiency of indirect selection (RSE) for improvement of DFY in evaluated genotypes of tall fescue in multiple harvest analysis

Variance component	DFY	H	CD	NS	FL
${\sigma}_A^2$	4350.66	75.60	12.61	71.22	9.50
${\sigma}_p^2$	19,102.97	223.52	30.22	189.56	31.22
${h}_n^2\pm \mathrm{S}E$	0.22 ± 0.04	0.32 ± 0.04	0.41 ± 0.04	0.37 ± 0.02	0.30 ± 0.03
R_y_	53.21	8.37	3.94	8.91	2.93
CR_y_	–	54.12	60.55	56.17	30.47
RSE	–	1.01	1.13	1.05	0.57

*DFY* dry forage yield (g/plant), *PH* plant height (cm/plant), *CD* crown diameter (cm/plant), *NS* number of stems per plant, *FL* flowering time, σ^2^_A_ and σ^2^_p_ additive and phenotypic variance, respectively, h^2^_n_ narrow sense heritability, *SE* standard error, *R* response to selection (%), *CR* correlated response to selection

Genetic correlation between traits ranged from 0.38 (between H and FL) to 0.86 (between DFY and H) (Table 5). The higher correlation values were obtained between DFY with H, CD, and NS. Flowering time (FL) had medium correlation with DFY (0.5), H (0.38), CD (0.44), and NS (0.36).

**Table 5:** Genetic correlation coefficients using best linear unbiased predictions (REML) for measured traits across three harvest and four years in the evaluated tall fescue germplasm

Traits	DFY	H	CD	NS	FL
DFY	1				
H	0.86 ± 0.09	1			
CD	0.85 ± 0.09	0.65 ± 0.11	1		
NS	± 0.12 0.83	± 0.10 0.69	0.59 ± 0.13	1	
FL	± 0.14 0.5	± 0.18 0.38	± 0.15 0.44	0.36 ± 0.19	1

*DFY* dry forage yield, *CD* Crown diameter, *H* Plant height, *NS* Number of stems per plant, *FO* flowering time

A broad range of breeding value was observed for all measured traits in both parental genotypes and their clonal progenies (Table S2 and S3). The breeding value for DFY varied from − 70.29 to 37.45 in parental genotypes and from − 45.79 to 159.90 in progenies (Table S2 and S3). In general, the highest values of DFY, H, CD, NS, and FL was observed in the parental genotypes 21 M and 1 M. However, genotypes 23 M, 25 L, and 20 L had also the high values of DFY, CD, and FL, respectively (Table S2). Parental genotypes 22 M and 3E had the lowest breeding values of DFY, H, and CD. Parental genotypes 2E and 2 L had the low breeding values of NS and parental genotypes 2E and 22 M had the low breeding values of FL (Table S2). In progenies, the highest breeding values of DFY was obtained in genotypes 154 and 133 (Table S3). Genotypes 133, 168, and 53 with high breeding values of H, and genotypes 135, 157, 53, and 167 with high breeding values of CD, and genotypes 133, 135, and 168 with high breeding values of NS, and genotypes 167, 154, 135, and 133 with high breeding values of FL were distinguished in the progenies (Table S3). Genotypes 65 and 127 had the lowest breeding values of DFY, H, CD, ND, and FL in the progenies.

**Fig. 2:**
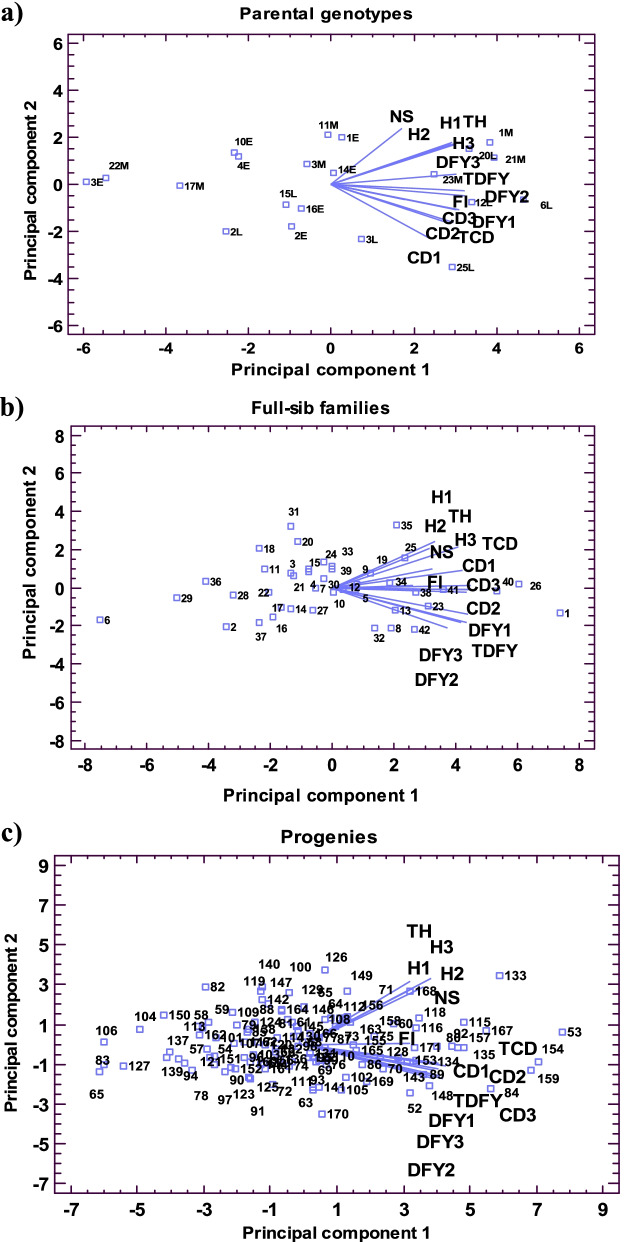
The biplot display of agro-morphological traits in parental genotypes (**a**), full-sib families (**b**), and progenies (**c**) of tall fescue. DFY1: spring forage yield, DFY2: summer forage yield, DFY3: autumn forage yield, TDFY: annual forage yield, H1: spring plant height, H2: summer plant height, H3: autumn plant height, TH: total plant height, CD1: spring crown diameter, CD2: summer crown diameter, CD3: autumn crown diameter, TCD: total crown diameter, NS: number of stems per plant, and FL: flowering time. Definition origin of the genotypes can be seen in Table 1

Principal component analysis (PCA) revealed that the first two components explained more than 83, 72, and 69% of the genetic variation in the parental genotypes, full-sib families, and individual progenies of tall fescue, respectively (Fig. 2). A broad range of variation was observed for the studied germplasm for all the evaluated traits across harvests. In all three groups (parental genotypes, full-sib families, and individual progenies) dry forage yield (DFY) and crown diameter (CD) at three harvest and flowering time most towards the variation in the first component, while plant height at three harvests (PH) and number of stems per plant (NS) contributed more towards second component. Therefore, selection based on moderate to high PC1 and PC2 value would lead to genotypes or families with favorable forge yield production and its related traits. In this respect, parental genotypes 23 M, 21 M, 20 L, and 1 M, and full-sib families 25, 26, 35, 40, and 41 and individual progenies 53, 60, 115, 116, 118, 133, 167, and 168 were identified as the superior genotypes. In contrast, parental genotypes 2 L, 17 M, 22 M, and 3E, and full-sib families 2, 6, 14, 16, 17, 27, and 37, and individual progenies 57, 65, 78, 83, 94, 127, 137, and 139 had low values of yield production and its related traits.

## Discussion

Yield performance, persistence or stability of grasses are greatly affected by climate conditions [27, 28]; therefore, it is important to assess genotypes during consecutive years and release cultivars with improved productivity and persistence. In breeding of perennial forage grasses, better understanding of the genetic variability and inheritance of economic traits are vital for identification and selection of preferable genotypes with possible utility in specific breeding programs such as developing productive varieties [9, 11, 15, 29]. In most studies in grass species, half-sib families derived from polycrosses are extensively used for genotypic evaluation, while full-sib families have been less considered in this regards [11, 13, 30]. Because, the level of self-incompatibility in grasses such as tall fescue is very high which limiting the construction of full-sib families. In our previous research, EST-SSR markers allowed to identify the paternal parents and then full-sib families in tall fescue, which considered as initial plant materials in the present study [26]. Remarkable genetic variation was observed between parental genotypes, full-sib families, and their progenies for yield performance and its related traits, emphasizing the high potential for genetic study of these traits and the possibility of selection superior genotypes for developing new varieties in the near future.

In both parental genotypes and full-sib families of tall fescue, summer dry forage yield (SUDFY) was found to be lower than spring (SPDFY) and autumn dry forage yield (AUDFY) during the years of experiment. Consistent with our finding, several researchers have reported the reduction of summer forage yield in some grass species such as tall fescue which is likely due to the higher temperature and induction of summer dormancy [31–33]. Kallida et al. [34] indicated that perennial grass species with summer dormancy have less yield production and growth during summer seasons than during spring and autumn seasons despite irrigation, and they have a better opportunity to survive and recover through periods of extended hot and dry conditions. However, we don’t have perfect information on summer dormancy of the studied germplasm, therefore; further experiments would be required to determine the level of this trait in this germplasm.

Estimation heritability and genetic correlation of traits is one of the main objectives in the plant breeding programs specially to identify an efficient approach for developing new varieties [35]. Generally, selection of plant for traits controlled by more than one gene is often a difficult task. On the other hand, usually the breeder goal is not to select an individual trait, but instead selecting number of traits simultaneously [12]. Therefore, knowledge about genetic correlation is very important, since it quantifies the value and direction of the influence of a specific trait on another and can assist selection [11, 36]. A positive genetic correlation between dry forage yield (DFY) with plant height (H), crown diameter (CD), number of stems per plant (NS), and flowering time (FL) indicates that selection based on higher H, CD, NS, and FL could lead to the selection of genotypes with better yield productivity. Similar to our findings, several researchers have been reported positive association between forage yield with yield related traits (such as H, CD, and NS) and flowering date in some perennial grass species [11, 14, 15, 18].

The estimates of narrow-sense heritability (h^2^_n_) for measured traits were higher in the multiple harvest analysis than the single harvest analysis, which could be due to the increasing the number of harvest. Consistent with our results, Acharya et al. [15] reported low values of h^2^_n_ for forage yield, plant height, and blooming through BLUP method in the single harvest analysis than in the multiple harvest analysis in alfalfa (*Medicago sativa* L.). Generally, estimation of h^2^_n_for seasonal and annual dry forage yield were lower than the yield related traits (such as H, CD, and NS) and flowering time (FL). Low values of h^2^_n_ for forage yield indicates that non-additive gene action may have a greater contribution to the expression of this economic trait, which implies lower odds of enhancement this trait through direct selection. Hence, indirect selection of the traits having higher h^2^_n_ values compared to yield productivity as well as those strongly associated with forage yield would be more efficient and promising [37]. Therefore, selection for high values of CD, H, NS, and FL (positively correlated with DFY) could be reliable and useful for achieving the indirect improvement of annual and seasonal forage yield. In both single and multiple harvest analysis, yield related traits (H, CD, and NS) had also high relative selection efficiency (RSE) (more than 1), confirming that selection for these traits would be more reliable and beneficial than direct selection for genetic improvement of DFY.

Although, different range of h^2^_n_has been reported for forage yield, its related traits, and flowering through different biometrical methods and designs, especially via half-sib families in different grass species, the range of estimated h^2^_n_ for agro morphological traits in this study through REML/BLUP analysis fall well within this range of values [13, 38–40]. However, still little literatures are available on genetic study through full-sib families in grasses. Low h^2^_n_for forge yield in the present study were comparable to those reported for alfalfa (0.08 to 0.37) [15], orchardgrass (0.16) [41], smooth bromegrass (0.20) [30], switchgrass (0.17–0.30) [42, 43], and tall fescue (0.18–0.45) [13, 44]. Acharya et al. [15] also reported low to moderate h^2^_n_ values for plant height (0.15–0.53) and flowering (0.08–0.51) in alfalfa during eleven harvests using BLUP procedure.

Prediction of breeding values is a prerequisite to successful implementation of long-term breeding programs. Breeding value refers to the property of an individual in a breeding population, which is mainly related to the additive genetic variance of a trait; therefore, is transmittable from parents to progenies and pertinent to the selection response [45, 46]. REML/BLUP analysis implemented in this study lead to identification of superior parental genotypes and progenies with high breeding values and great potential for the simultaneous improvement for the measured traits. This facilitates the selection of candidate parents of crosses for developing synthetic varieties or hybrids with high heterosis in future programs.

The principal component analysis (PCA) is one of the most successful multivariate techniques used for screening suitable genotypes [47]. Wide distribution of parental genotypes, full-sib families, and individual progenies on the biplots of PCA, indicated a broad range of variation for the studied germplasm for all the evaluated traits across harvests. Based on the biplots of PCA, the favorable parental genotypes, full-sib families, and individual progenies with better forage yield production and its related traits were distinguished. Generally, based on the breeding values, biplot of PCA, and stability parameter, the parental genotypes 21 M, 1 M, and 20 L and the full-sib families 26 (♀21 M × ♂11 M), 41 (♀20 L × ♂4E), and 25 (♀21 M × ♂1 M) were recognized as suitable and most stable genotypes. In contrast, parental genotypes 22 M and 3E and full-sib families 6 (♀3E × ♂1 M), 2 (♀1E × ♂4E), 29 (♀22 M × ♂10E), and 36 (♀6 L × ♂1E) were recognized as inferior and unstable genotypes. Among the studied progenies, genotypes 53 (♀1E × ♂2E), 133 (♀21 M × ♂1 M), 135 (♀21 M × ♂11 M), 154 (♀12 L × ♂2 L), 167 (♀20 L × ♂4E), and 168 (♀20 L × ♂4E) were identified as preferable and best hybrid combination and genotypes 127 (♀17 M × ♂1 M) and 65 (♀3E × ♂1 M) were detected as unfavorable hybrid combination. It is remarkable that most of the superior full-sib families and progenies were derived from the crosses of the superior genotypes 21 M, 1 M, and 20 L as maternal parents with other genotypes as paternal parent. Therefore, some part of this superiority can be due to the direct effect of the cytoplasm and the mitochondrial genes of the maternal parent as well as the interaction between the nuclear genes and the cytoplasm of the maternal parent, which need to further experiments for demonstrating [48].

## Conclusions

In conclusion, wide range of genetic variability for forage yield, yield related traits, and flowering time points to the high potential of the studies germplasm for genetic improvement of these traits through full-sib mating in tall fescue. The narrow-sense heritability of seasonal and annual dry forage yield was lower than the narrow-sense heritability of yield related traits (such as H, CD, and NS) and FL, indicates that non-additive gene action may play a major role in the genetic control of forage yield (DFY) which led to lower odds of enhancement this trait through direct selection. Positive genetic correlation between H, CD, NS, and FL with dry forage yield, also high relative selection efficiency of yield related traits (more than 1) suggest that these traits could be used for enhancing DFY through indirect selection. The REML/BLUP was an adequate method for estimating the heritability and detecting the superior parental genotypes and progenies with higher breeding values for future breeding program in tall fescue. Parental genotypes 21 M, 1 M, and 20 L were identified as superior and stable genotypes, which can be used in breeding programs for developing synthetic varieties. These genotypes could also produce the best full-sib families or hybrid combinations (such as ♀21 M × ♂1 M, ♀21 M × ♂11 M, ♀12 L × ♂2 L, ♀20 L × ♂4E) when they were mostly used as maternal parent.

## Methods

### Experimental site

This research was conducted at the research farm of the College of Agriculture, Isfahan University of Technology, Isfahan, Iran (32° 30′ N, 51° 20′ E, 1630 m asl). The soil at the site was clay loam (pH 7.5) with an average bulk density of 1.48 g/cm^3^ in the top 60 cm layer of the soil profile. The average annual precipitation and temperature were 122 mm and 17 °C, respectively.

### Plant materials, field management, and measurements

The primary plant materials included of 21 genotypes of tall fescue (Table 1) which were chosen from a broad base germplasm collection according to various agro-morphological, physiological and root traits and used as parents for crossing [11, 26]. In order to generate a reference breeding population, these genotypes were crossed following a polycross design. As a results, 960 progenies (from 21 half-sib families) were obtained and evaluated for agro-morphological traits [26]. From these 960 progenies, 120 genotypes genotyped using diagnostic EST-SSR primers in the previous study [26]. These 120 genotypes which were divided in to 42 full-sib families along with the 21 parental genotypes were used as the plant material in the present study. Identification of the tall fescue genotypes used in this study has been done in the botanical laboratory of Isfahan University of Technology (IUT). A voucher specimen of this material has been deposited in a publicly available herbarium of IUT (Deposition number: 36594).

This germplasm was evaluated in the field under normal irrigation condition according to the randomized complete block design with two replications during 2017–2020. The clone of each genotype was space planted in the field with inter-row and intra-row spacing 50 and 45 cm, respectively. Plants were irrigated using a surface drip tape irrigation system. No limitation of irrigation was conducted during the whole experiment. Irrigation was applied when 45% of the total available water was depleted from the root-zone to maintain the soil water content at the field capacity [49].

The above-ground biomass (forage) was harvested manually three times in each year. The first harvest was in late spring after flowering, the second and third one was in late summer and autumn to assess complete growth, respectively. At each harvest, the grass was cut from 5 cm above the ground and the weight of dry forage yield per plant was recorded after drying at 72 °C for 48 h. The annual dry forage yield of each year (ADFY) was calculated by the sum of the spring (SPDFY), summer (SUDFY) and autumn (AUDFY) forage yield. Number of stems per plant (NS), plant height (H), crown diameter (CD), and flowering time (FL) were measured as recommended by Pirnajmedin et al. [7].

### Statistical analysis

Data (residuals) were tested for normality using the Kolmogorov-Smirnov test; subsequently, the analysis of variance (ANOVA) was performed using the PROC Mixed by repeated measures in SAS software (v9.2) [50]. The means were compared using the Fisher’s LSD test (*P* < 0.05). Stability analysis was calculated for forage yield using the stability parameter proposed by Eberhart and Russell [51]. The stability parameter was the regression coefficient of the average forage yield of each family in each year on the average of all families in each year.

The pedigree of all the families were known and the recode of genotypes was done using CFC software. The pedigree information in BLUP analysis was used for constructing the relationship matrix and then estimating the genetic parameters and predicting the breeding values, which is done by the DMU software [52]. The first analysis was performed by individual harvest and then a multi-harvest model was fitted. Year and cut were treated as fixed and genotype was treated as random effects. All analyses were conducted using the mixed linear model given by Henderson as follow [53]:

1 $$Y= X\beta + Zu+e$$

$$\left[\begin{array}{cc}{X}^{\prime }{R}^{-1}X& {X}^{\prime }{R}^{-1}Z\\ {}{Z}^{\prime }{R}^{-1}X& {Z}^{\prime }{R}^{-1}Z+{G}^{-1}\end{array}\right]\left[\begin{array}{c}\hat{\beta}\\ {}\hat{\mathrm{u}}\end{array}\right]=\left[\begin{array}{c}{X}^{\prime }{R}^{-1}y\\ {}{Z}^{\prime }{R}^{-1}y\end{array}\right]$$

where, Y is the vector of observation, β and u are vectors of fixed and random effects, respectively, X and Z are the associated design matrices, and e is a random residual vector. The random effects are assumed to be distributed as u ~ *MVN* (0, G) and e ~ *MVN* (0, R), where *MVN* (u, V) denotes the multivariate normal distribution with mean vector u and variance-covariance matrix V. The G is the genetic variance/covariance matrix and R is the residual variance/covariance matrix.

Individual harvest analysis was performed using the model:

2 $$Y=\mu + Xy+Z\mathrm{g}+e$$

where, the u is the overall mean; X and Z represent the incidence matrices for fixed and random effects, respectively; y is the fixed effect for year; g is the random vector of genotype, g ~ *MVN* (0, A*σ*^2^_g_), which *σ*^2^_g_ is variance of genotype and A is a relationship (kinship) matrix; e is the random vector of error, e ~ *MVN* (0, I*σ*^2^_e_), which *σ*^2^_e_ is the variance of residual and I is an identity matrix of its proper size.

Multiple harvest analysis was performed using the model:

3 $$Y=\mu + Xc+ Xy+Z\mathrm{g}c+ Wp+e$$

where, the u is the overall mean; where, the u is the overall mean; c is the fixed vector of cut; y is the fixed vector of year; gc is the random vector of genotype within each cut, gc ~ *MVN* (0, GA), which G is the genetic variance/covariance matrix and A is a relationship (kinship) matrix; p is the random vector of the permanent environment, p ~ *MVN* (0, I *σ*^2^_e_); e is the random vector of error within each cut, e ~ *MVN* (0, RI), which *σ*^2^_e_ is the variance of residual, R is the residual variance/covariance matrix, and I is an identity matrix of its proper size. *X*, *Z*, and W represent the incidence matrices for these effects.

Variance component and narrow sense heritability of traits were estimated using the restricted maximum likelihood (REML/BLUP) analysis by DMUAI procedure and breeding values (BVi) were computed by DMU4 procedure in DMU software, respectively [52]. The narrow sense heritability was estimated by dividing of additive genetic variance to phenotyping variance by the following formula:

4 $${h}_{n=}^2\frac{\sigma_a^2}{\sigma_g^2+{\sigma}_p^2+{\sigma}_e^2}$$

where *σ*2*a*, *σ*2*g*, *σ*2*p*, *σ*2*e* are the additive genetic variance, genotypic variance, permanent environment variance, and residual variance, respectively.

Using the bivariate analysis, the genetic correlations for each pair of traits were estimated from the genetic variance-covariance matrices from the model described above.

Relative selection efficiency (RSE) for improvement of dry forage yield (DFY) were estimated as described by Falconer and Mackay [45] and Searle [54] by the following formula:

5 $$\mathrm{RSE}=\frac{{\mathrm{CR}}_{\mathrm{y}}}{{\mathrm{R}}_{\mathrm{y}}}=\frac{\mathrm{i}\times \mathrm{rg}\times \mathrm{hx}\times \mathrm{hy}\times \upsigma \mathrm{p}\left(\mathrm{y}\right)}{\mathrm{i}\times \times {h}_x^2\times \upsigma \mathrm{p}\left(\mathrm{y}\right)}$$

where CR_y_ is correlated response to selection, Ry is response to selection, i is the selection intensity of 10% (1.75), h^2^_x_ is heritability of trait x, σ_p(x)_ is the square root of genotypic variance of trait x, r_g_ is the genotypic correlation coefficient between two traits, h_x_ and h_y_ are the root square of narrow-sense heritability of traits of x and y, respectively. The correlated trait (y) is dry forage yield and RSE was only calculated based on dry forage yield.

Principal component analysis (PCA) was performed based on correlation matrix to reduce the multiple dimensions of data space using SAS (Proc princomp), and biplots were drawn using Stat Graphics statistical software [55].

We confirm that all methods complied with relevant institutional, national, and international guidelines and legislation.

## Supplementary Information

**Additional file 1.**

## Data Availability

The data sets supporting the results of this article are included within the article and its additional files.

## Abbreviations

REML/BLUP: Restricted maximum likelihood/Best linear unbiased prediction
ADFY: Annual dry forage yield
SPDFY: Spring dry forage yield
SUDFY: Summer dry forage yield
AUDFY: Autumn dry forage yield
NS: Number of stems per plant (NS)
H: Plant height
CD: Crown diameter
FL: Flowering time
PCA: Principal component analysis
