# Supplementary material for: Genetic parameters and selection in full-sib families of tall fescue using best linear unbiased prediction (BLUP) analysis
Source: BMC Plant Biol. 2022 Jun 14;22:293. doi: 10.1186/s12870-022-03675-w (PMC9199132; doi:10.1186/s12870-022-03675-w)
Supplement: Supplementary file 1 — Additional file 1. [file 12870_2022_3675_MOESM1_ESM.doc]

| **Table S1** Analysis of variance for measured traits in parental genotypes (G) and full-sib families (F) of tall fescue assessed at three harvests (spring, summer and autumn) during 2017-2020 | | | | | | |
| --- | --- | --- | --- | --- | --- | --- |
| **Mean square** | | | | | | |
| FL | NS | CD | H | DFY | Degree of freedom | Source of variation |
| **Parental genotype** | | | | | | |
| 24987.76^**^ | 286234.10^**^ | 10457.67^**^ | 71220.51^**^ | 49999.50^**^ | 3 | Y |
| 60.02 | 498.83 | 301.02 | 2169.52 | 2230.54 | 4 | Rep(Y) |
| 542.23^**^ | 9874.45^**^ | 296.99^**^ | 2894.32^**^ | 7550.65^**^ | 20 | G |
| 208.49^**^ | 7542.31^**^ | 35.42^**^ | 524.19^**^ | 931.17^**^ | 60 | G*Y |
| 43.99 | 344.91 | 17.74 | 156.45 | 324.49 | 80 | Rep*G(Y) |
| - | - | 625.31^**^ | 18985.74^**^ | 16787.52^**^ | 2 | Har |
| - | - | 5.37^**^ | 137.21^*^ | 145.99^*^ | 40 | Har*G |
| - | - | 2130.24^**^ | 7988.64^**^ | 35987.91^**^ | 6 | Har*Y |
| - | - | 8.01^**^ | 69.27^ns^ | 20114.10^**^ | 120 | Har*G*Y |
| - | - | 2.31 | 62.49 | 81.15 | 168 | Error |
| **Full-sib families** | | | | | | |
| 28542.24^**^ | 328674.12^**^ | 11987.98^**^ | 27563.52^**^ | 3541681.25^**^ | 3 | Y |
| 310.54 | 1947.01 | 301.12 | 1984.79 | 189954.10 | 4 | Rep(Y) |
| 396.75^**^ | 2421.78^**^ | 641.21^**^ | 4553.30^**^ | 94345.78^**^ | 41 | F |
| 200.12^**^ | 942.88^**^ | 88.35^**^ | 289.04^*^ | 22347.07^**^ | 123 | F*Y |
| 24.86 | 298.99 | 14.79 | 145.01 | 2497.89 | 164 | Rep*F(Y) |
| - | - | 789.34^**^ | 24150.24^**^ | 425897.78^**^ | 2 | Har |
| - | - | 15.37^**^ | 101.04^**^ | 2248.98^*^ | 82 | Har*F |
| - | - | 1736.21^**^ | 1021.14^**^ | 459971.43^**^ | 6 | Har*Y |
| - | - | 12.87^**^ | 38.97^ns^ | 2141.51^**^ | 246 | Har*F*Y |
| - | - | 5.47 | 28.33 | 1343.27 | 336 | Error |
| *: Significant at the 0.05 probability level, **: Significant at the 0.01 probability level, ns: non-significant.  Rep: replication, Y: year, G: parental genotypes, H: harvest, F: full-sib families  DFY: dry forage yield, H: plant height, CD: crown diameter, NS: number of stems per plant, FL: Flowering time | | | | | | |

| **Table S2** Breeding values of 21 parental genotypes for measured traits | | | | | |
| --- | --- | --- | --- | --- | --- |
| FL | NS | CD | H | DFY | Genotype name |
| -0.82 | 0.55 | -2.22 | 1.94 | -47.43 | 1E |
| -2.58 | -11.91 | -0.81 | -10.87 | -41.32 | 2E |
| -0.8 | -10.72 | -5.88 | -17.6 | -69.30 | 3E |
| -1.36 | -10 | -3.14 | -5.17 | -63.26 | 4E |
| -1.23 | -1.19 | -2.8 | -7.28 | -68.12 | 10E |
| -0.38 | -7.15 | -2.4 | -4.17 | -40.72 | 14E |
| -1.48 | -9.82 | -0.75 | -8.91 | -47.00 | 16E |
| 1.3 | 3.05 | 1.16 | 5.72 | 21.12 | 1M |
| -0.3 | -4.29 | -2.03 | -4.66 | -50.08 | 3M |
| -1.59 | 0.16 | -1.9 | 0.34 | -59.80 | 11M |
| -2.01 | -11.43 | -3.79 | -12.9 | -58.11 | 17M |
| 1.35 | 6.07 | 1.18 | 3.15 | 37.45 | 21M |
| -2.24 | -10.77 | -5.2 | -15.99 | -70.26 | 22M |
| 0.14 | -8.56 | -1.16 | 0.34 | 23.51 | 23M |
| 0.41 | -11.63 | -1.15 | -13.62 | -65.71 | 2L |
| 0.21 | -8.47 | 0.41 | -9.29 | 4.63 | 3L |
| 0.68 | -6.9 | 0.39 | 0.07 | 9.59 | 6L |
| 1.05 | -3.55 | 0.31 | 0.48 | 14.23 | 12L |
| 0.11 | -9.4 | -1.87 | -9.14 | -54.61 | 15L |
| 1.48 | 2.11 | -1.04 | 0.37 | 16.35 | 20L |
| 0.41 | -9.49 | 2.34 | -7.01 | 14.91 | 25L |

| **Table S3** Breeding values of 120 clone of progenies for measured traits | | | | | | |
| --- | --- | --- | --- | --- | --- | --- |
| Progeny code | Parental plants | DFY | H | CD | NS | FL |
| 53 | ♀1E × ♂2E | 139.73 | 14.01 | 5.32 | 3.98 | -0.25 |
| 52 | ♀1E × ♂2E | 88.03 | 5.38 | 3.94 | 3.93 | 0.11 |
| 54 | ♀1E × ♂4E | 53.95 | 3.68 | -1.6 | -1.14 | -1.26 |
| 60 | ♀1E × ♂1M | 18.51 | 8.7 | 3.72 | 1.77 | -0.26 |
| 57 | ♀1E × ♂1M | -14.02 | 0.71 | 0.3 | -5.02 | -0.64 |
| 59 | ♀1E × ♂1M | -7.42 | 4.23 | -0.2 | -3.93 | -0.76 |
| 58 | ♀1E × ♂1M | -19.71 | 3.68 | 0.3 | -3.71 | -1.57 |
| 55 | ♀1E × ♂1M | 8.32 | 8.02 | 1.22 | 1.37 | -0.58 |
| 56 | ♀1E × ♂1M | 11.51 | 2.33 | 1.74 | 0.63 | -0.08 |
| 61 | ♀3E × ♂10E | -15.29 | 4.67 | 1.21 | -4.65 | 1.21 |
| 62 | ♀3E × ♂10E | -7.15 | 2.11 | 0.61 | -3.34 | 2.58 |
| 63 | ♀3E × ♂14E | 24.63 | 0.49 | 3.2 | -4.11 | 0.09 |
| 64 | ♀3E × ♂14E | 4.81 | 5.73 | 2.32 | 3.08 | 0.83 |
| 65 | ♀3E × ♂1M | -45.79 | -3.79 | -3.79 | -6.31 | -5 |
| 68 | ♀3E × ♂11M | 3.89 | 3.83 | 3.31 | -3.35 | 1.33 |
| 66 | ♀3E × ♂11M | 3.77 | 2.39 | 2.8 | -2.03 | 1.15 |
| 67 | ♀3E × ♂11M | 16.12 | 1.4 | 2.27 | -1.4 | 0.4 |
| 69 | ♀4E × ♂11M | 92.77 | 4.72 | 1.69 | 0.33 | -0.82 |
| 70 | ♀4E × ♂11M | 117.5 | 6.18 | 2.47 | 5.18 | -1.69 |
| 71 | ♀4E × ♂17M | 47.01 | 8.8 | 1.85 | 2.94 | -0.39 |
| 73 | ♀4E × ♂17M | 32.11 | 7.98 | 2.28 | 2.03 | -3.53 |
| 72 | ♀4E × ♂17M | 23.71 | 1.88 | 2.85 | -4.02 | -3.1 |
| 75 | ♀10E × ♂1M | 74.15 | 7.68 | 1.35 | 7.23 | -2.88 |
| 77 | ♀10E × ♂1M | 20.76 | 4.49 | 1.84 | 11.69 | 1.16 |
| 78 | ♀10E × ♂1M | -5.39 | 0.26 | 0.62 | 5.46 | -1.31 |
| 74 | ♀10E × ♂1M | 26.12 | 2.06 | 1.52 | 10.95 | -1.26 |
| 76 | ♀10E × ♂1M | 35.12 | 3.36 | 2.6 | 5.75 | -0.26 |
| 79 | ♀14E × ♂1E | 2.06 | 3.9 | 0.16 | 2.42 | 1.53 |
| 80 | ♀14E × ♂2E | 85.12 | 9.33 | 3.55 | 18.99 | -0.64 |
| 89 | ♀14E × ♂2E | 123.49 | 7.42 | 2.84 | 10.53 | -1.64 |
| 86 | ♀14E × ♂2E | 85.14 | 5.67 | 2.26 | 17.9 | 0.16 |
| 87 | ♀14E × ♂2E | 36.9 | 5.7 | 2.94 | 11.27 | 0.22 |
| 84 | ♀14E × ♂2E | 121.72 | 7.69 | 4.9 | 18.36 | -0.64 |
| 85 | ♀14E × ♂2E | 15.35 | 5.12 | 1.57 | 5.96 | -0.83 |
| 81 | ♀14E × ♂2E | 13.66 | 7.16 | 2.26 | 6.65 | -1.39 |
| 88 | ♀14E × ♂2E | 13.67 | 5.63 | 1.43 | 9.9 | 0.66 |
| 90 | ♀14E × ♂2E | 14.5 | 1.87 | 1.85 | 10.42 | -0.77 |
| 82 | ♀14E × ♂2E | 11.83 | 7.75 | -0.84 | 8.65 | -1.02 |
| 83 | ♀14E × ♂2E | -18.83 | -1.64 | 0.06 | 2.53 | 0.78 |
| 92 | ♀14E × ♂25L | 106.8 | 8.68 | 2.35 | 5.64 | -2.47 |
| 91 | ♀14E × ♂25L | 38.46 | 0.26 | 0.96 | 7.79 | -0.47 |
| 97 | ♀14E × ♂11M | 19.53 | -1.03 | 0.3 | 7.78 | 2.18 |
| 93 | ♀1M × ♂11M | 47.28 | -0.25 | 2.38 | 10.81 | 1.18 |
| 96 | ♀1M × ♂11M | 13.7 | 0.44 | 1.26 | 4.75 | 1.43 |
| 95 | ♀1M × ♂11M | 32.51 | 2.59 | 2.36 | 6.06 | 0.68 |
| 94 | ♀1M × ♂11M | 20.54 | -1.32 | -0.44 | 4.86 | 0.75 |
| **Table S3** continued | | | | | | |
| Progeny code | Parental plants | DFY | H | CD | NS | FL |
| 98 | ♀1M × ♂11M | 32.63 | 2.48 | 1.67 | 6.75 | 2.81 |
| 99 | ♀1M × ♂21M | 22.8 | 3.08 | 0.95 | 10.58 | 1.53 |
| 100 | ♀1M × ♂21M | -19.6 | 7.21 | 0.2 | 6.81 | 2.65 |
| 101 | ♀1M × ♂21M | -35.95 | 1.88 | 0.39 | -0.04 | 3.46 |
| 102 | ♀3M × ♂2E | 103.28 | 3.89 | 0.93 | 4.11 | -0.17 |
| 104 | ♀3M × ♂2E | 19.48 | 2.29 | -1.5 | 1.94 | -0.98 |
| 103 | ♀3M × ♂2E | 64.4 | 3.71 | -0.52 | 1.26 | -0.42 |
| 108 | ♀3M × ♂1M | 38.71 | 6.29 | -0.59 | 3.73 | 0.03 |
| 105 | ♀3M × ♂1M | 78.46 | 2.01 | 0.16 | 1.5 | -1.49 |
| 107 | ♀3M × ♂1M | 10.68 | 1.93 | -1.42 | 0.64 | -0.49 |
| 106 | ♀3M × ♂1M | -13.87 | -1.05 | -1.12 | -2.72 | -1.37 |
| 109 | ♀3M × ♂6L | -18.48 | 3.32 | -1.33 | 1.2 | 0.42 |
| 115 | ♀11M × ♂2E | 69.26 | 11.71 | 4.4 | 19.81 | 1.02 |
| 118 | ♀11M × ♂2E | 45.74 | 10.52 | 4.3 | 12.33 | -0.16 |
| 111 | ♀11M × ♂2E | 28.66 | 4.92 | 2.96 | 8.04 | 0.96 |
| 112 | ♀11M × ♂2E | 44.13 | 9.68 | 2.44 | 8.16 | 0.71 |
| 110 | ♀11M × ♂2E | 22.97 | 5.85 | 3.94 | 11.13 | 1.27 |
| 114 | ♀11M × ♂2E | 42.6 | 6.25 | 1.08 | 11.87 | 0.96 |
| 113 | ♀11M × ♂2E | 9.64 | 5.12 | 0.11 | 8.67 | 0.33 |
| 117 | ♀11M × ♂2E | 9.92 | 4.59 | 1.86 | 8.38 | 2.33 |
| 116 | ♀11M × ♂2E | 63.48 | 10.07 | 3.83 | 12.9 | 0.21 |
| 119 | ♀11M × ♂16E | -1.28 | 8.06 | -0.19 | -0.62 | 0.34 |
| 120 | ♀11M × ♂1M | -13.82 | 4.46 | 1.5 | 3.77 | -1.29 |
| 126 | ♀17M × ♂1M | -1.12 | 10.47 | 0.46 | -2.69 | -0.51 |
| 121 | ♀17M × ♂1M | 14.18 | 0.93 | -0.77 | -2.69 | -0.58 |
| 124 | ♀17M × ♂1M | 8.79 | 4.67 | -0.45 | -1.2 | -0.89 |
| 125 | ♀17M × ♂1M | -2.81 | 0.15 | 1.42 | -1.49 | -1.01 |
| 122 | ♀17M × ♂1M | 47.67 | 3.12 | 0.32 | 2.05 | -0.14 |
| 123 | ♀17M × ♂1M | -4.9 | 0.04 | 0.42 | 4.62 | -2.63 |
| 127 | ♀17M × ♂1M | -37.11 | -2.54 | -2.67 | -7.81 | -3.6 |
| 128 | ♀17M × ♂2L | 88.46 | 5.87 | 2.11 | 7.93 | 0.56 |
| 129 | ♀21M × ♂14E | 33.26 | 8 | 1.63 | 8.3 | 0.17 |
| 130 | ♀21M × ♂14E | 31.07 | 6.3 | 2.85 | 5.27 | -0.32 |
| 133 | ♀21M × ♂1M | 155.43 | 16.11 | 4.41 | 25.77 | 3.54 |
| 132 | ♀21M × ♂1M | 14.4 | 5.36 | 2.31 | 4.16 | -0.66 |
| 131 | ♀21M × ♂1M | 29.16 | 6.13 | 2.82 | 4.39 | -0.1 |
| 134 | ♀21M × ♂11M | 117.25 | 8.67 | 3.94 | 7.58 | 0.86 |
| 135 | ♀21M × ♂11M | 75.26 | 10.29 | 5.84 | 24.21 | 3.67 |
| 136 | ♀21M × ♂6L | 22.92 | 3.32 | 1.88 | 3.46 | 1.13 |
| 137 | ♀22M × ♂4E | 5.64 | 0.12 | -1.95 | 16.29 | -0.91 |
| 138 | ♀22M × ♂4E | 7.2 | 3.88 | -0.82 | 9.95 | 1.67 |
| 139 | ♀22M × ♂10E | -34.009 | -1.16 | -1.07 | 2.99 | -1.72 |
| 140 | ♀22M × ♂1M | -19.06 | 7.22 | -1.14 | 12.54 | -0.75 |
| 141 | ♀22M × ♂1M | 43.6 | 1.62 | 0.58 | 15.91 | -2.13 |
| 142 | ♀22M × ♂21M | -29.6 | 7.31 | -0.17 | 6.47 | -0.62 |
| 144 | ♀23M × ♂14E | 74.91 | 2.83 | 0.24 | 2.12 | 1.13 |
| 143 | ♀23M × ♂14E | 108.95 | 6.76 | 2.28 | 7.49 | 1.88 |
| 146 | ♀23M × ♂17M | 28.89 | 7.13 | 1.22 | 5.79 | -1.45 |
| 145 | ♀23M × ♂17M | 15.57 | 7.22 | 1.64 | 7.11 | -2.2 |
| 147 | ♀23M × ♂3L | 10.189 | 8.64 | 0.24 | 5.1 | -0.2 |
| **Table S3** continued | | | | | | |
| Progeny code | Parental plants | DFY | H | CD | NS | FL |
| 148 | ♀23M × ♂3L | 111.23 | 5.98 | 2.74 | 21.39 | -1.51 |
| 149 | ♀23M × ♂15L | 35.61 | 10.71 | 0.35 | 20.2 | 1.94 |
| 151 | ♀6L × ♂1E | -21.81 | -1.37 | 0.37 | -6.24 | 3.71 |
| 150 | ♀6L × ♂1E | -22.94 | 0.94 | -1.84 | -1.06 | 2.33 |
| 152 | ♀6L × ♂1M | 8.26 | -0.68 | -0.61 | -1.85 | 2.02 |
| 155 | ♀12L × ♂1E | 110.84 | 8.09 | 2.11 | 15.94 | 1.27 |
| 157 | ♀12L × ♂1E | 65.15 | 9.21 | 5.4 | 13.82 | 1.22 |
| 158 | ♀12L × ♂1E | 40.24 | 8.73 | 3.17 | 4.56 | 2.03 |
| 159 | ♀12L × ♂1E | 54.04 | 11.1 | 4.88 | 7.36 | 2.46 |
| 160 | ♀12L × ♂1E | 26.95 | 3.19 | 2.49 | 1.36 | 2.53 |
| 161 | ♀12L × ♂1E | 23.33 | 2.75 | 3.22 | 2.79 | 2.84 |
| 164 | ♀12L × ♂1M | 19.44 | 7.63 | 1.51 | 8.51 | 0.78 |
| 162 | ♀12L × ♂1M | 15.85 | 4.23 | 0.1 | 5.77 | 0.9 |
| 165 | ♀12L × ♂1M | 66.76 | 7.94 | 2.91 | 3.77 | 0.65 |
| 163 | ♀12L × ♂1M | 49.39 | 9.6 | 2.18 | 7.66 | 0.34 |
| 156 | ♀12L × ♂2L | 75.92 | 10.54 | 2.52 | 15.48 | 1.73 |
| 153 | ♀12L × ♂2L | 90.95 | 7.68 | 3.96 | 13.76 | 2.61 |
| 154 | ♀12L × ♂2L | 159.09 | 8.72 | 3.12 | 21.83 | 3.74 |
| 167 | ♀20L × ♂4E | 130.74 | 12.24 | 5.29 | 5.64 | 4.1 |
| 168 | ♀20L × ♂4E | 111.69 | 15.15 | 1.45 | 23.16 | -0.9 |
| 169 | ♀20L × ♂4E | 126.74 | 6.01 | 2.21 | 3.13 | 0.46 |
| 166 | ♀20L × ♂4E | 65.09 | 7.68 | 1.85 | 2.9 | 1.65 |
| 170 | ♀20L × ♂14E | 114.68 | 0.7 | 3.15 | 4.49 | 0.43 |
| 171 | ♀20L × ♂14E | 95.83 | 8 | 4.43 | 4.26 | 0.99 |
